# Activation-Induced Marker Assay to Identify and Isolate HCV-Specific T Cells for Single-Cell RNA-Seq Analysis

**DOI:** 10.3390/v16101623

**Published:** 2024-10-17

**Authors:** Mohamed Eisa, Nicol Flores, Omar Khedr, Elsa Gomez-Escobar, Nathalie Bédard, Nourtan F. Abdeltawab, Julie Bruneau, Arash Grakoui, Naglaa H. Shoukry

**Affiliations:** 1 Centre de Recherche du Centre Hospitalier de l’Université de Montréal (CRCHUM), Tour Viger, Local R09.414, 900 rue St-Denis, Montréal, QC H2X 0A9, Canadanourtan.abdeltawab@pharma.cu.edu.eg (N.F.A.);; 2 Département de Microbiologie, Infectiologie et Immunologie, Université de Montréal, Montréal, QC H3C 3J7, Canada; 3 Department of Microbiology and Immunology, Faculty of Pharmacy, Cairo University, Cairo 3296121, Egypt; 4 School of Pharmacy, Newgiza University, Giza 3296121, Egypt; 5 Département de Médecine Familiale et Département d’Urgence, Université de Montréal, Montréal, QC H3C 3J7, Canada; 6 Department of Medicine, Emory University, Atlanta, GA 30322, USA; 7 Département de Médecine, Université de Montréal, Montréal, QC H3C 3J7, Canada

**Keywords:** hepatitis C virus, AIM assay, antigen-specific T cells, single-cell RNA sequencing

## Abstract

Identification and isolation of antigen-specific T cells for downstream transcriptomic analysis is key for various immunological studies. Traditional methods using major histocompatibility complex (MHC) multimers are limited by the number of predefined immunodominant epitopes and MHC matching of the study subjects. Activation-induced markers (AIM) enable highly sensitive detection of rare antigen-specific T cells irrespective of the availability of MHC multimers. Herein, we have developed an AIM assay for the detection, sorting and subsequent single-cell RNA sequencing (scRNA-seq) analysis of hepatitis C virus (HCV)-specific T cells. We examined different combinations of the activation markers CD69, CD40L, OX40, and 4-1BB at 6, 9, 18 and 24 h post stimulation with HCV peptide pools. AIM^+^ CD4 T cells exhibited upregulation of CD69 and CD40L as early as 6 h post-stimulation, while OX40 and 4-1BB expression was delayed until 18 h. AIM^+^ CD8 T cells were characterized by the coexpression of CD69 and 4-1BB at 18 h, while the expression of CD40L and OX40 remained low throughout the stimulation period. AIM^+^ CD4 and CD8 T cells were successfully sorted and processed for scRNA-seq analysis examining gene expression and T cell receptor (TCR) usage. scRNA-seq analysis from this one subject revealed that AIM^+^ CD4 T (CD69^+^ CD40L^+^) cells predominantly represented Tfh, Th1, and Th17 profiles, whereas AIM^+^ CD8 T (CD69^+^ 4-1BB^+^) cells primarily exhibited effector and effector memory profiles. TCR analysis identified 1023 and 160 unique clonotypes within AIM^+^ CD4 and CD8 T cells, respectively. In conclusion, this approach offers highly sensitive detection of HCV-specific T cells that can be applied for cohort studies, thus facilitating the identification of specific gene signatures associated with infection outcome and vaccination.

## 1. Introduction

T cells play a central role in the adaptive immune response to pathogens, tumors and vaccines. Quantification of antigen-specific T-cell responses has been traditionally performed by assessing cytokine production, cell proliferation and/or staining with fluorescently conjugated peptide/human leukocyte antigen (HLA) multimers [1]. However, these assays have limitations in capturing the full spectrum of antigen-specific T cells. For instance, proliferation assays may overlook cells with reduced proliferation capacities, such as Tregs. These assays are also susceptible to bystander effects, where IL-2 produced by stimulated antigen-specific T cells can trigger the proliferation of nearby non-specific T cells [1]. Moreover, the use of fluorescently labelled peptide–MHC multimers to identify antigen-specific T cells is hindered by the complexity of identifying the immunodominant peptides and their restricted MHC molecules matching a diverse study population.

Staining for activation-induced markers (AIM) allows the detection and phenotyping of antigen-specific T cells using flow cytometry. This assay measures the upregulation of specific cell surface markers following stimulation of peripheral blood mononuclear cells (PBMCs) with whole antigens or peptide pools which are processed and presented by autologous antigen-presenting cells without the need for prior determination of HLA type and immunodominant epitopes. Numerous markers are upregulated by T cells upon T cell receptor (TCR)-dependent stimulation compared to unstimulated cells. These markers include CD69, CD40L (CD154), OX40 (CD134), 4-1BB (CD137), and IL-2Rα (CD25). CD69 has been used to identify antigen-specific CD8 T cells upon TCR stimulation, with peak expression at 18 h [2]. However, expression of CD69 could also be triggered by TCR-independent stimulation through IL-2, IL-4, IL-12, interferon (IFN)-α, IFN-γ, and α4β1 integrin, highlighting the importance of combining it with other marker(s) [1,2,3]. Indeed, co-expression of CD69 and 4-1BB was used to detect SARS-CoV-2 specific T cells [4,5] and CD69/CD40L co-expression defined HIV-specific CD4 T cells [6]. CD40L is a member of the tumor necrosis factor (TNF) superfamily and can be transiently expressed by T cells (and mainly expressed on activated CD4 T cells) in response to antigen stimulation [7,8]. Depending on the antigen used, CD40L^+^ T cells can secrete large amounts of cytokines including IL-2, IL-4, IL-5, IL-10, IFN-γ, and TNF [9,10]. The surface expression of CD40L is downregulated at 6 h post activation, probably due to endocytosis after binding with its cognate receptor, CD40 [11]. To overcome this downregulation, anti-CD40L antibody or anti-CD40 blocking antibodies can be added for the duration of activation [9,10]. OX40 is another member of the TNF superfamily and has been used in combination with CD25 to identify antigen-specific CD4 T cells [12], with a peak expression at 48 h post-stimulation [13]. Recently, OX40, alone or in combination with other activation markers, has been used to identify antigen-specific CD4 T cells [4,6,14]. Finally, 4-1BB, another TNF superfamily member, was used in combination with CD69 to identify antigen-specific CD8 T cells [15], with optimal expression at 24 h post-activation [16].

In the present study, we examined the kinetics of expression of different activation markers and optimized an 18-h AIM assay to simultaneously detect HCV-specific CD4 and CD8 T cells. Our assay successfully enabled FACS sorting for downstream transcriptional profiling and TCR clonotypes of AIM^+^ cells at the single-cell level.

## 2. Materials and Methods

### 2.1. Study Subject

An HCV resolver who cleared two subsequent infections was recruited from the Montreal Hepatitis C cohort (HEPCO) of people who inject drugs. The study was approved by the Research Ethics Committee of the Centre de Recherche du Centre hospitalier de l’Université de Montréal (CRCHUM) (Approval number: SL 05.014). The participant provided written informed consent to participate in this study. The subject was HIV-negative. PBMCs collected during acute HCV reinfection (180 days post estimated date of infection) were used through all experiments.

### 2.2. Activation-Induced Markers Assay

Cryopreserved PBMCs were thawed and washed twice with RPMI-5 (RPMI medium supplemented with 5% human serum (Wisent, Saint-Jean-Baptiste, QC, Canada) and 1% penicillin/streptomycin (Wisent)). Cells (average 2.5 × 10^6^) were then incubated in 1 mL of RPMI-5 for 3 h at 37 °C, 5% CO_2_ in 5 mL polypropylene snap cap tubes (Corning, Tamaulipas, Mexico), followed by the addition of 5 µL of CD40 blocking antibody (Miltenyi Biotec, Gladbach-Ehrenfeld, Deutschland, Germany) to prevent the interaction of CD40L with CD40 and its subsequent downregulation. Cells were then stimulated for 6, 9, 18 or 24 h at 37 °C, 5% CO_2_ with 1 µg/mL of HCV overlapping peptides pool representing non-structural protein NS5B (obtained through BEI Resources, NIAID, NIH: Peptide Array, Hepatitis C Virus, H77, NS5B Protein, Catalog No: NR-3756). Cells stimulated with 1 µg/mL staphylococcal enterotoxin B (SEB) (Toxin Technology Inc., Sarasota, FL, USA) and unstimulated cells served as positive and negative controls, respectively. Cells were then collected and transferred to a 96-well V-bottom plate, washed twice with FACS buffer (PBS 1×, 1% FBS, 0.01% sodium azide), and stained for 30 min at 4 °C with surface markers (see Table 1 for antibodies) and viability dye [LIVE/DEAD cell stain kit (Thermo Fisher Scientific, Saint-Laurent, QC, Canada)]. Cells were then washed twice and fixed using 1% formaldehyde (Millipore Sigma, Oakville, ON, Canada). Multiparameter flow cytometry was performed at the flow cytometry core of the CRCHUM using a BD Fortessa instrument equipped with five lasers (UV [355 nm], violet [450 nm], blue [488 nm], yellow–green [561 nm] and red [640 nm]) and FACSDiva version 9.2 (BD Biosciences, Milpitas, CA, USA). FCS data files were analyzed by using FlowJo (version 10.8.1 for Mac; BD Biosciences).

### 2.3. Single-Cell RNA Sequencing

AIM^+^ CD4 (CD69^+^ CD40L^+^; total: 5250 cells) and AIM^+^ CD8 T cells (CD69^+^ 4-1BB^+^; total: 40,800 cells) were FACS-sorted at 18 h post-stimulation with HCV non-structural protein (NS5B) peptides pool and loaded (1722 CD4 and 4671 CD8 T cells) on a Chromium Controller (10X Genomics, Pleasanton, CA, USA). Single-cell RNA-seq libraries were prepared according to the 10X Genomics chromium single-cell protocol for Next GEM Single Cell 5′ Reagent Kits v2 (Dual Index). Quality control and quantification of libraries were performed using the Agilent Bioanalyzer 2100 system (Agilent Technologies, Santa Clara, CA, USA). Libraries were loaded onto an Illumina NovaSeq PE100—2500 M read (Illumina Inc., San Diego, CA, USA). Sequencing was performed by the Genome Quebec Innovation Center (Montreal, QC, Canada). Cell Ranger (10X Genomics, version 7.2.0) was used to generate gene-barcode matrices. All scRNA-seq analyses were performed in R (version 4.3.2) using the package Seurat (version 5.0.3) [17]. To filter out doublets and dead cells, the number of detected genes per cell and percent mitochondrial genes were plotted and outliers were removed (i.e., cells with detected genes fewer than 500 or more than 4000 and cells having over 5% mitochondrial genes). Principal component analysis was performed and the top 20 principal components were used for UMAP analysis. Single-cell TCR sequences were mapped and combined using scRepertoire [18].

### 2.4. Statistics

Statistical analyses were performed with Prism version 10.1.0 (GraphPad). Details of tests are provided in the figure legends. *p* values less than 0.05 were considered significant.

## 3. Results

### 3.1. HCV-Specific CD4 T Cells Exhibit Upregulation of CD69, CD40L and OX40

**Figure 1:**
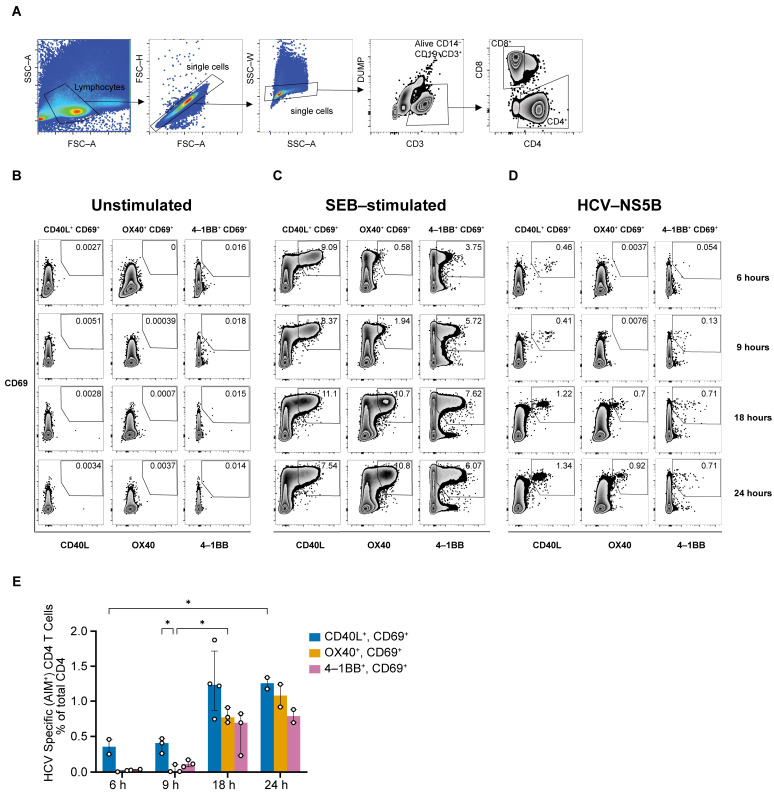
Expression of CD69, CD40L and OX40 at 18 h defines AIM^+^ CD4 T cells. (**A**) Representative gating strategy used for the detection of AIM^+^ cells among total CD4^+^ and CD8^+^ T cells. (**B**–**D**) Representative staining for different AIM marker pairs. PBMC from a resolver of HCV reinfection were stimulated with either: (**B**) no peptide (unstimulated control), (**C**) staphylococcal enterotoxin B (SEB) (positive control), or (**D**) HCV non-structural protein (NS5B; aa 2708–3014) peptides pool for 6, 9, 18, or 24 h followed by the detection of AIM markers upregulation in CD4^+^ T cells using flow cytometry. AIM^+^ CD4^+^ T cells are defined by the expression of CD69 in conjunction with either CD40L, OX40, or 4.1BB and presented as a percentage of total CD4^+^ T cells. (**E**) Bar charts depicting the frequencies of AIM^+^ CD4^+^ T cells at the indicated time points post-stimulation. Data points represent different experimental replicates and bars represent median with interquartile range after background staining subtraction from unstimulated samples. Statistical analysis was performed using a two-way ANOVA followed by Tukey’s multiple-comparison posttest. * = *p* < 0.05.

We investigated the kinetics of expression of various activation-induced markers, including CD69, CD40L, OX40, and 4-1BB, on CD4 T cells in response to a peptide pool of HCV non-structural protein 5B (NS5B; aa: 2708–3014; genotype 1a H77 sequence). PBMCs obtained from an HCV resolver who successfully cleared two consecutive episodes of HCV infection were stimulated with either no peptides (unstimulated control), SEB (positive control) or the NS5B peptide pool for 6, 9, 18, or 24 h, and the upregulation of the AIM markers was assessed by flow cytometry (Figure 1A–D). A representative gating strategy is presented in Figure 1A. CD4 T cells expressing CD69 and CD40L were detectable as early as 6 h post-stimulation (Figure 1D), with a median frequency of 0.35% of total CD4^+^ T cells. The median frequencies of CD69^+^ CD40L^+^ CD4 T cells increased to 1.2% and 1.3% at 18 and 24 h, respectively (Figure 1E). In contrast, the expression of both OX40 and 4-1BB was delayed and observed only at 18 h post-stimulation. The median frequency of CD4^+^ CD69^+^ OX40^+^ T cells was 0.8% and 1.2% at 18 and 24 h, respectively, while the median frequency of CD4^+^ CD69^+^ 4-1BB^+^ T cells was 0.7% and 0.8% at 18 and 24 h, respectively (Figure 1E). These findings suggest that the combined expression of CD69, CD40L, and OX40 at 18 h could be used to define HCV-specific CD4 T cells.

### 3.2. Upregulation of CD69 and 4-1BB Defines HCV-Specific CD8 T Cells

**Figure 2:**
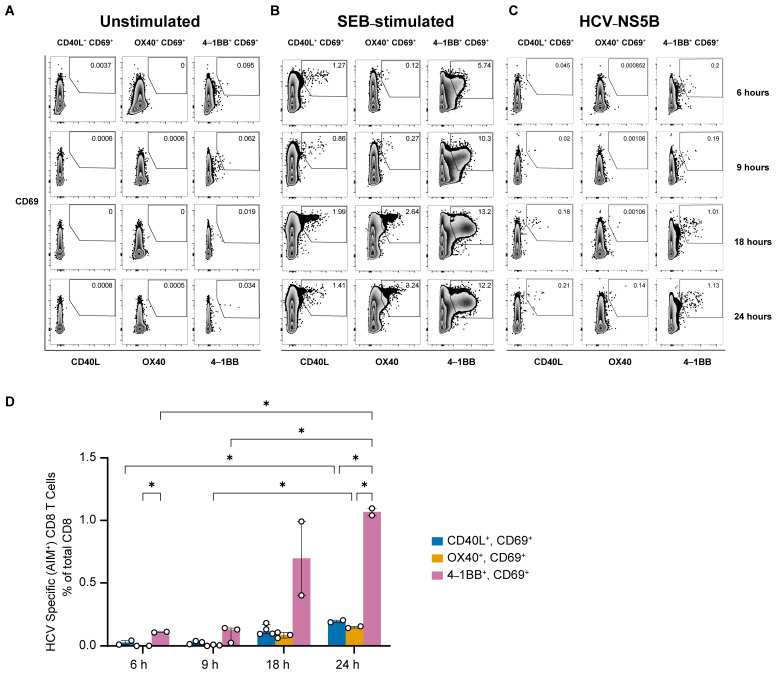
AIM^+^ CD8^+^ T cells primarily express CD69 and 4-1BB at 18 h post-activation. (**A**–**C**) Representative staining demonstrating the expression of various AIM marker pairs by CD8^+^ T cells. PBMC from a resolver of HCV reinfection were stimulated with either: (**A**) no peptide (unstimulated control), (**B**) staphylococcal enterotoxin B (SEB) (positive control), or (**C**) HCV non-structural protein (NS5B; aa 2708–3014) peptides pool for 6, 9, 18, or 24 h followed by the detection of AIM markers upregulation in CD8^+^ T cells using flow cytometry. Cells were gated as in Figure 1A. AIM^+^ CD8^+^ T cells are defined by the coexpression of CD69 and 4.1BB and presented as a percentage of total CD8^+^ T cells. (**D**) Bar charts depicting the frequencies of AIM^+^ CD8^+^ T cells at the indicated time points post-stimulation. Data points represent different experimental replicates and bars represent median with interquartile range after background staining subtraction from unstimulated samples. Two-way ANOVA followed by Tukey’s multiple-comparison posttest. * = *p* < 0.05.

To identify AIM^+^ CD8 T cells, we assessed the expression of the four AIM markers on CD8 T cells at 6, 9, 18 and 24 h post-stimulation with either no peptides (unstimulated control), SEB (positive control) or the NS5B peptide pool for 6, 9, 18, or 24 h, and the upregulation of the AIM markers was assessed by flow cytometry (Figure 2A–C). AIM^+^ CD8 T cells exhibited delayed expression of 4-1BB that was detectable only 18 h post-stimulation. The median frequencies of CD8^+^ CD69^+^ 4-1BB^+^ T cells were 0.7% and 1.1% at 18 and 24 h post-stimulation, respectively. The frequency of CD8^+^ T cells expressing both CD69 and 4-1BB was significantly higher at 24 h compared to 6 and 9 h, with no significant difference between 18 and 24 h (Figure 2D). However, CD8 T cells expressing CD69 plus either CD40L or OX40 were scarcely detected, with median frequency as low as 0.2% at 24 h post-stimulation. These results suggest that the combined expression of CD69 and 4-1BB at 18 h could reliably define HCV-specific CD8^+^ T cells.

### 3.3. scRNA Seq Defined Multiple Distinct Subsets of AIM^+^ CD4 T Cells

**Figure 3:**
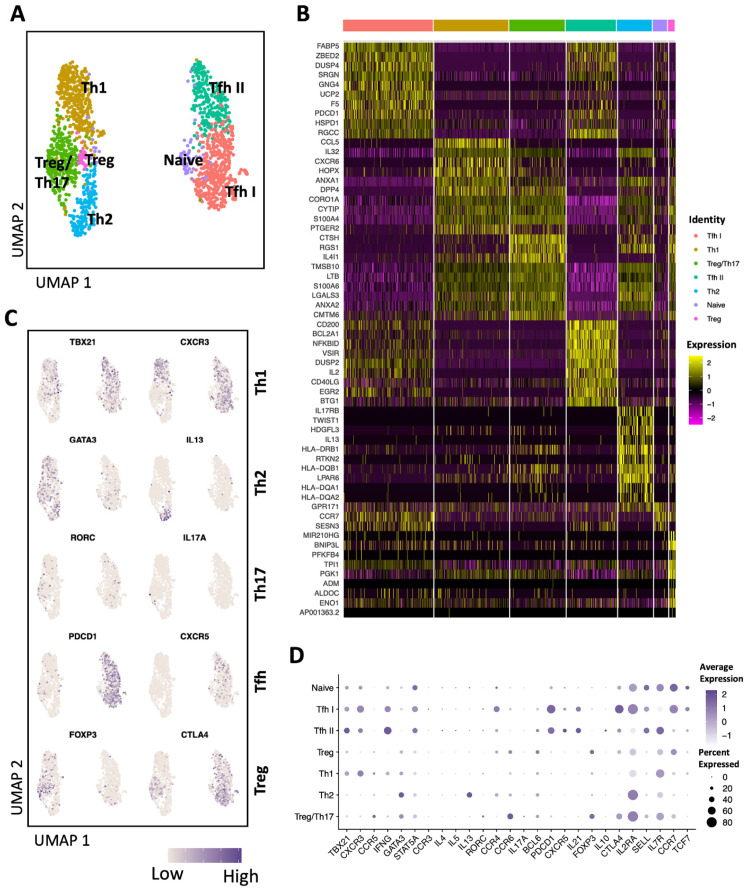
Gene expression analysis of AIM^+^ CD4 T cells. (**A**) Uniform Manifold Approximation and Projection (UMAP) plot of scRNA-seq data from AIM^+^ CD4 T cells that FACS sorted from an HCV-resolver subject. (**B**) Heatmap showing the expression of the top marker genes of each cluster. (**C**) Feature plots displaying the expression of representative markers of different CD4 subsets. (**D**) Dot plot presenting the expression of canonical CD4 markers in each cluster. Color represents the average expression and dot size represents the percentage of cells that express the gene.

To assess whether AIM+ T cells are suitable for downstream transcriptomic analysis of HCV-specific T cells, AIM^+^ (CD69^+^ CD40L^+^) CD4 T cells were FACS sorted and processed for scRNA-seq. In this subject, we observed the expansion of seven distinct clusters of AIM^+^ CD4 T cells, as visualized by Uniform Manifold Approximation and Projection (UMAP) (Figure 3A). A complete list of expressed genes and their statistical significances are listed in Supplementary Table S1. Analysis of top differentially expressed genes per cluster (Figure 3B, Supplementary Table S1) and selected canonical markers for different CD4 T cells subsets (Figure 3C,D) indicated that the primary expanding clusters were identified as Tfh I, Th1, Treg/Th17, Tfh II, and Th2, while two minor clusters represented Tregs and naïve-like AIM^+^ CD4. Interestingly, HCV-specific Tfh and naïve CD4 T cells clustered separately from Th1, Th2, Treg, and Treg/Th17. AIM^+^ Tfh I cluster constituted the largest expanding subset and exhibited expression of lineage gene markers characteristic of Tfh cells, including *CXCR5*, *PDCD1*, *BCl6*, *CD200,* and *IL-21*, along with markers of Th1 such as *CXCR3*, *TBX21* and *IFNG,* and Th2 *CCR4* (Figure 3B–D). The AIM^+^ Tfh II cluster showed upregulation of Th2 marker *CCR4* in addition to Tfh markers (*CXCR5*, *PDCD1*, *BCl6*, *CD200,* and *IL-21*), although *GATA3* and *IL-13* were barely detectable in both Tfh clusters (Figure 3C,D). Compared to Tfh I, the Tfh II subset preferentially upregulated (Figure 3B) genes belonging to the early response to TCR engagement, including *NR4A1* (encoding Nur77), *NR4A3*, *DUSP2*, *BTG1*, and *EGR1* [19,20], inflammation-associated genes (*NFKBID*), and V-domain immunoglobulin suppressor of T cell activation (VISTA) encoded by *VISR*, which regulates CD4 T cell quiescence and peripheral tolerance [21]. Other differentially expressed genes between Tfh I and Tfh II subsets included *CD200, BCL2A1, IL2, CD40L, CD82, PGAM1, TNFSF8, CCR7, IL2RA, TCF7,* and *CTLA4* (Figure 3B,D). Th1 cells constituted the second largest expanding subset and were characterized by the expression of *CXCR3*, *TBX21*, *IL7R*, *IL2RA,* and *IFNG* (Figure 3C,D). Treg/Th17 cells displayed upregulation of the transcription factor RAR-related orphan receptor C (*RORC*) and the chemokine receptor *CCR6*, which are principal phenotypic markers of human Th17 cells [22,23]. Moreover, this subset additionally expressed the Treg marker *FOXP3* (Figure 3C,D). Both Th1 and Treg/Th17 subsets have the *ANXA1* gene, encoding the anti-inflammatory protein Annexin-1, which is critical for T cell activation and differentiation [24] among the top differentially expressed genes (Figure 3B). Treg/Th17 exhibited higher expression of *CCR6*, *CCR5, CTLA4,* and *IL2RA* compared to the Treg subset (Figure 3D). The Th2 cell cluster showed upregulation of the markers *GATA3* and *IL-13*, which are hallmark genes for Th2 (Figure 3C,D). Collectively, our scRNA-seq data indicate that sorting AIM^+^ CD4 T cells facilitated the detection of different HCV-specific cells clusters with varying profiles, including the Tfh, Th1, Th17, and Treg subsets.

### 3.4. AIM+ CD8 T Cells Have Mainly Effector and Memory Signature

**Figure 4:**
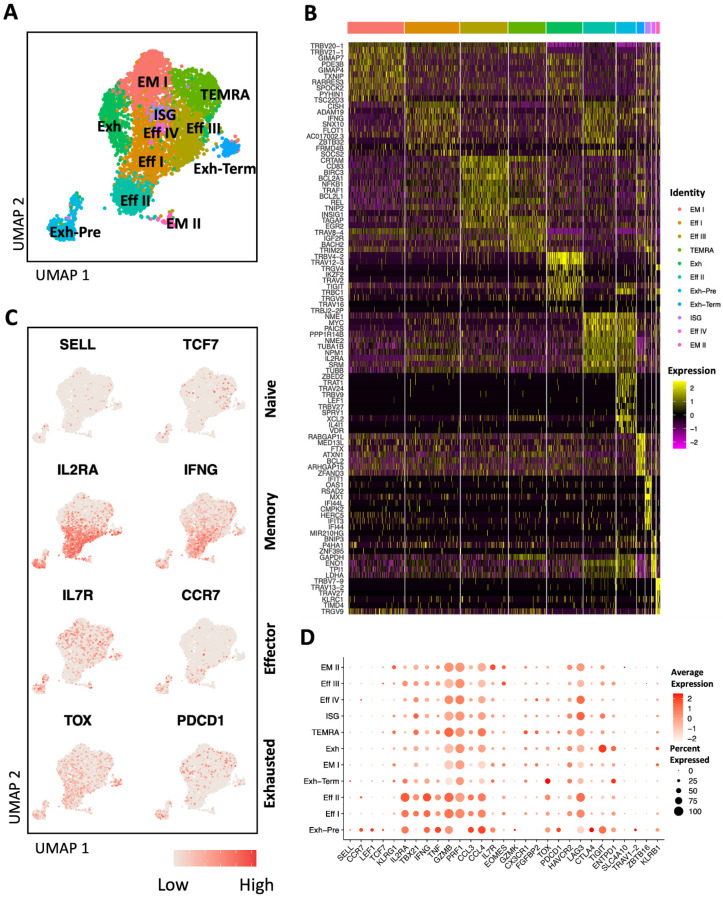
Gene expression analysis of AIM^+^ CD8 T cells. (**A**) Uniform Manifold Approximation and Projection (UMAP) plot of scRNA-seq data from AIM^+^ CD8 T cells derived from an HCV-infected subject. (**B**) Heatmap showing the expression of the top marker genes of each cluster. (**C**) Feature plots displaying the expression of representative markers of different CD8 subsets. (**D**) Dot plot presenting the expression of canonical CD8 markers in each cluster. Color represents the average expression and dot size represents the percentage of cells that express the gene. Eff: Effector, EM: Effector memory, Exh: Exhausted, Exh-Pre: Precursor exhausted, Exh-Term: Terminally exhausted, ISG: Interferon-stimulated genes, TEMRA: Terminally differentiated EM.

AIM^+^ (CD69^+^ 4-1BB^+^) CD8 T cells were also sorted to study their transcriptomic profile by scRNA-seq. A complete list of expressed genes and their statistical significances are listed in Supplementary Table S2. Analysis of the top differentially expressed genes per cluster and selected canonical markers for different CD8 T cells subsets (Figure 4A–D, Supplementary Table S2) unveiled four distinct clusters of effector/cytotoxic *IL2RA^+^*, *IL7R^−^*, *CCR7*^−^ CD8 T cells (Eff I, Eff II, Eff III, and Eff IV), expressing the cytotoxic molecules (*GZMB* and *PRF1*) and cytokines (*IFNG*, *TNF*, *CCL3,* and *CCL4*). Eff I and Eff II showed a higher expression of *IFNG* and the transcription factor *ZBTB32* compared to the other effector populations. *ZBTB32* is a key regulator of effector versus memory responses in CD8 T cells [25]. The Eff III cluster was characterized by the expression of the late-stage activation marker *CRTAM* [26], while Eff IV was characterized by the expression of the metabolic gene *GAPDH* (Figure 4A–D). Additionally, AIM^+^ CD8 T cells comprised two distinct *IL7R^+^*, *CCR7*^−^, and *EOMES*^+^ effector memory clusters (EM I and EM II). Terminally differentiated effector memory CD8 (TEMRA) cells expressing *CX3CR1* and *FGFBP2* represented one cluster. In addition to the effector cells, the AIM assay enabled the capture of different stages of CD8 T cells exhaustion, including precursor-exhausted (Exh-Pre *TCF7^+^*), exhausted (Exh *TCF7^−^*) and terminally exhausted (Exh-Term *ENTPD1^+^*) subsets. Exhausted AIM^+^ CD8 T cells exhibited variable upregulation of different exhaustion markers, including *TOX*, *PDCD1*, *TIGIT*, *CTLA4,* and *LAG3*. These findings underscore the efficacy of the AIM assay in capturing HCV-specific CD8 T cells with distinct activation and exhaustion states.

### 3.5. AIM Assay Captures T Cell Receptor (TCR) Diversity of HCV-Specific CD4 and CD8 T Cells

**Figure 5:**
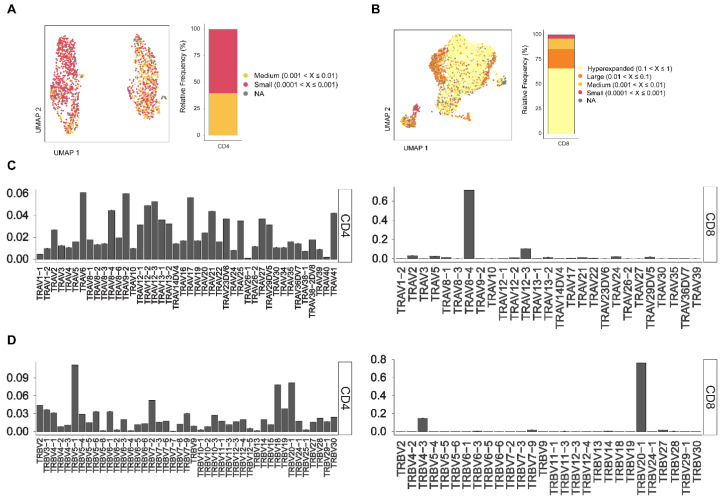
T cell receptor analysis of AIM^+^ CD4 and CD8 T cells. (**A**,**B**) Uniform Manifold Approximation and Projection (UMAP) plots and bar charts showing the relative frequency of TCR clonotypes within the indicated categories in (**A**) AIM^+^ CD4 T cells and (**B**) AIM^+^ CD8 T cells. (**C**,**D**) Bar charts depicting the usage of (**C**) T-cell receptor alpha variable region (TRAV) and (**D**) T-cell receptor beta variable region (TRBV) genes in AIM^+^ CD4 T cells (**left**) and AIM^+^ CD8 T cells (**right**).

Next, we tested the capacity of the AIM assay to identify different T cell receptor clonotypes by performing TCR analysis to investigate the distribution of unique clonotypes among the AIM^+^ CD4 and CD8 T cells. In this subject, the AIM^+^ CD4 repertoire was composed of 1023 unique clonotypes, while the AIM^+^ CD8 repertoire was less diverse, as it was composed of 160 unique clonotypes. The individual clonotype distribution within the total repertoire was further stratified into four categories as follows: small (0.0001 < X ≤ 0.001), medium (0.001 < X ≤ 0.01), large (0.01 < X ≤ 0.1), and hyperexpanded (0.1 < X ≤ 1). Within the AIM^+^ CD4 T cells, the majority (60.24%) of the repertoire was composed of unique small-size clonotypes, while 39.75% were medium-size clonotypes (Figure 5A). In contrast AIM^+^ CD8 showed less diversity, with 66.14% of the total repertoire composed of hyperexpanded clonotypes, 19.4% large, 10.68% medium, and 3.8% small-size clonotypes (Figure 5B). Furthermore, the analysis of the usage of T-cell receptor alpha variable region (TRAV) and beta variable region (TRBV) genes in AIM^+^ CD4 and CD8 T cells indicated a dominant usage of TRAV8-4 and TRBV20-1 regions by AIM^+^ CD8 clones, in contrast to AIM^+^ CD4 cells, which did not have specific dominant usage of both regions and confirming the more focused nature of AIM^+^ CD8 TCR clonotypes in this subject (Figure 5C,D).

## 4. Discussion

In this study, we optimized an AIM assay utilizing a combination of activation markers to detect HCV-specific T cells with minimal background in the unstimulated condition. This optimized assay facilitated the simultaneous detection and sorting of HCV-specific CD4 and CD8 T cells which were subsequently processed for downstream scRNA sequencing analysis for both gene expression and TCR clonotyping. To enhance the assay’s sensitivity, we employed a set of four AIM markers: CD69, CD40L, OX40, and 4-1BB. We demonstrated that the combination of CD69 with CD40L and/or OX40 effectively identified HCV-specific CD4 T cells, while HCV-specific CD8 T cells were predominantly identified by the coexpression of CD69 and 4-1BB. Early upregulation of CD69 and CD40L on HCV-specific CD4 T cells was observed within 6 to 9 h post-stimulation, aligning with previous results from 9 h AIM assays used to detect SARS-CoV-2 and HIV-specific CD4 T cells [6,27,28,29,30]. However, AIM^+^ CD4 T cells expressing CD69 in combination with OX40 or 4-1BB were only detectable at late time points (18–24 h post-stimulation), which is consistent with optimal upregulation of OX40 observed at 18–24 h [6,31,32,33,34]. Indeed, the 44 to 48 h AIM assay has been employed to detect OX40 expressing CD4 T cells in response to trial vaccines against *Escherichia coli* [35] and HIV [36,37]. AIM^+^ CD8 T cells were primarily defined by the late upregulation of CD69/4-1BB (18–24 h post-stimulation), which is similar to reported results for the detection of SARS-CoV-2 -specific CD8 T cells [5,15].

Investigation of HCV-specific T-cell immune responses has primarily been conducted using peptide–MHC class I and II tetramer or multimer staining for detection of antigen-specific CD8 [38,39,40,41] and CD4, respectively [42,43,44,45]. However, constructing MHC class II multimers poses significant challenges and limited yield [46], thereby restricting the detection of HCV-specific CD4 T cells. Moreover, peptide–multimer staining requires prior identification of immunodominant epitopes and characterization of patients’ MHC alleles, which limit the detectable repertoire of antigen-specific T cells. Our optimized assay addresses these limitations by enabling the simultaneous sorting and analysis of both HCV-specific CD4 and CD8 T cells from the same sample, irrespective of MHC background, thus providing a more comprehensive understanding of HCV-specific immune responses in a diverse population and upon infection with diverse viral variants. It will be interesting in future studies to compare the gene expression profile of cells isolated using MHC multimers versus the AIM assay.

Although the AIM assay provides a reliable alternative to conventional assays such as proliferation and cytokine production, it remains a functional assay that is dependent on TCR-mediated cell activation. This might suggest that naïve and exhausted cells could be overlooked. However, our single-cell data identified naïve-like CD4 and terminally exhausted CD8 T cell subsets. These findings underscore the concept that the AIM assay enables the detection of various activation and exhaustion states in T cells which cannot be captured by proliferation and cytokines production assays. It is important to note that some of these markers maybe indicative of activation rather than exhaustion, since the sample tested was collected during the acute phase of infection in a subject that successfully resolved HCV. Additional studies examining the capacity to detect exhausted HCV-specific T cells at later time points, especially when cells drop in frequency or become severely exhausted, are warranted.

## 5. Conclusions

We developed a robust assay that enables co-assessment and sorting of HCV-specific CD4 and CD8 T cells in a single panel, which is followed by scRNA sequencing and TCR clonotyping. This approach detected different polarization states of CD4 T cells in addition to effector, memory, and exhausted CD8 T cells subsets. Future work should investigate whether different combinations of AIM markers may enrich antigen-specific CD4 and/or CD8 T cells with specific phenotypes, signatures, or skewed cytokine production.

## Figures and Tables

**Table 1:** Flow cytometry antibodies.

Antigen	Fluorophore	Clone	Catalog Number	Supplier
CD14	V500	M5E2	561391	BD Biosciences
CD19	V500	HIB19	561121	BD Biosciences
CD3	BUV496	UCHT1	612940	BD Biosciences
CD4	BV605	RPA-T4	562658	BD Biosciences
CD8	APC-H7	SK1	560179	BD Biosciences
CD69	PE	FN50	555531	BD Biosciences
CD154 (CD40L)	BUV737	TRAP1	748983	BD Biosciences
CD134 (OX40)	FITC	ACT35	555837	BD Biosciences
CD137 (4-1BB)	PE Dazzle 594	4B4-1	309826	BioLegend

## Data Availability

RNA sequencing data are available at the Gene Expression Omnibus (GEO) under accession number GSE273224 and can be reviewed using the following token: irujgkcixhyzvkt. Analysis scripts are available on Github at: https://github.com/shoukryLab/Eisa_et_al_2024 (accessed on 14-October 2024).

## Supplementary Materials

The following supporting information can be downloaded at: https://www.mdpi.com/article/10.3390/v16101623/s1, Table S1: Complete gene expression list for AIM+ CD4 T cells; Table S2: Complete gene expression list for AIM+ CD8 T cells.

## References

[1] Poloni C., Schonhofer C., Ivison S., Levings M.K., Steiner T.S., Cook L. (2023). T-cell activation-induced marker assays in health and disease. Immunol. Cell Biol..

[2] Wu H., Witzl A., Ueno H. (2019). Assessment of TCR signal strength of antigen-specific memory CD8^+^ T cells in human blood. Blood Adv..

[3] Cimo A.M., Ahmed Z., McIntyre B.W., Lewis D.E., Ladbury J.E. (2013). CD25 and CD69 induction by α4β1 outside-in signalling requires TCR early signalling complex proteins. Biochem. J..

[4] Altosole T., Rotta G., Uras C.R.M., Bornheimer S.J., Fenoglio D. (2023). An optimized flow cytometry protocol for simultaneous detection of T cell activation induced markers and intracellular cytokines: Application to SARS-CoV-2 immune individuals. J. Immunol. Methods.

[5] Grifoni A., Weiskopf D., Ramirez S.I., Mateus J., Dan J.M., Moderbacher C.R., Rawlings S.A., Sutherland A., Premkumar L., Jadi R.S. (2020). Targets of T Cell Responses to SARS-CoV-2 Coronavirus in Humans with COVID-19 Disease and Unexposed Individuals. Cell.

[6] Reiss S., Baxter A.E., Cirelli K.M., Dan J.M., Morou A., Daigneault A., Brassard N., Silvestri G., Routy J.P., Havenar-Daughton C. (2017). Comparative analysis of activation induced marker (AIM) assays for sensitive identification of antigen-specific CD4 T cells. PLoS ONE.

[7] Meier S., Stark R., Frentsch M., Thiel A. (2008). The influence of different stimulation conditions on the assessment of antigen-induced CD154 expression on CD4^+^ T cells. Cytometry A.

[8] Chattopadhyay P.K., Yu J., Roederer M. (2006). Live-cell assay to detect antigen-specific CD4^+^ T-cell responses by CD154 expression. Nat. Protoc..

[9] Frentsch M., Arbach O., Kirchhoff D., Moewes B., Worm M., Rothe M., Scheffold A., Thiel A. (2005). Direct access to CD4^+^ T cells specific for defined antigens according to CD154 expression. Nat. Med..

[10] Chattopadhyay P.K., Yu J., Roederer M. (2005). A live-cell assay to detect antigen-specific CD4^+^ T cells with diverse cytokine profiles. Nat. Med..

[11] Yellin M.J., Sippel K., Inghirami G., Covey L.R., Lee J.J., Sinning J., Clark E.A., Chess L., Lederman S. (1994). CD40 molecules induce down-modulation and endocytosis of T cell surface T cell-B cell activating molecule/CD40-L. Potential role in regulating helper effector function. J. Immunol..

[12] Zaunders J.J., Munier M.L., Seddiki N., Pett S., Ip S., Bailey M., Xu Y., Brown K., Dyer W.B., Kim M. (2009). High levels of human antigen-specific CD4^+^ T cells in peripheral blood revealed by stimulated coexpression of CD25 and CD134 (OX40). J. Immunol..

[13] Gramaglia I., Weinberg A.D., Lemon M., Croft M. (1998). Ox-40 ligand: A potent costimulatory molecule for sustaining primary CD4 T cell responses. J. Immunol..

[14] Dan J.M., Lindestam Arlehamn C.S., Weiskopf D., da Silva Antunes R., Havenar-Daughton C., Reiss S.M., Brigger M., Bothwell M., Sette A., Crotty S. (2016). A Cytokine-Independent Approach To Identify Antigen-Specific Human Germinal Center T Follicular Helper Cells and Rare Antigen-Specific CD4^+^ T Cells in Blood. J. Immunol..

[15] Rydyznski Moderbacher C., Ramirez S.I., Dan J.M., Grifoni A., Hastie K.M., Weiskopf D., Belanger S., Abbott R.K., Kim C., Choi J. (2020). Antigen-Specific Adaptive Immunity to SARS-CoV-2 in Acute COVID-19 and Associations with Age and Disease Severity. Cell.

[16] Wölfl M., Kuball J., Eyrich M., Schlegel P.G., Greenberg P.D. (2008). Use of CD137 to study the full repertoire of CD8^+^ T cells without the need to know epitope specificities. Cytometry A.

[17] Satija R., Farrell J.A., Gennert D., Schier A.F., Regev A. (2015). Spatial reconstruction of single-cell gene expression data. Nat. Biotechnol..

[18] Borcherding N., Bormann N.L., Kraus G. (2020). scRepertoire: An R-based toolkit for single-cell immune receptor analysis. F1000Research.

[19] Zemmour D., Zilionis R., Kiner E., Klein A.M., Mathis D., Benoist C. (2018). Single-cell gene expression reveals a landscape of regulatory T cell phenotypes shaped by the TCR. Nat. Immunol..

[20] Wakamatsu E., Mathis D., Benoist C. (2013). Convergent and divergent effects of costimulatory molecules in conventional and regulatory CD4^+^ T cells. Proc. Natl. Acad. Sci. USA.

[21] ElTanbouly M.A., Zhao Y., Nowak E., Li J., Schaafsma E., Le Mercier I., Ceeraz S., Lines J.L., Peng C., Carriere C. (2020). VISTA is a checkpoint regulator for naïve T cell quiescence and peripheral tolerance. Science.

[22] Annunziato F., Cosmi L., Santarlasci V., Maggi L., Liotta F., Mazzinghi B., Parente E., Filì L., Ferri S., Frosali F. (2007). Phenotypic and functional features of human Th17 cells. J. Exp. Med..

[23] Acosta-Rodriguez E.V., Rivino L., Geginat J., Jarrossay D., Gattorno M., Lanzavecchia A., Sallusto F., Napolitani G. (2007). Surface phenotype and antigenic specificity of human interleukin 17-producing T helper memory cells. Nat. Immunol..

[24] D’Acquisto F., Merghani A., Lecona E., Rosignoli G., Raza K., Buckley C.D., Flower R.J., Perretti M. (2007). Annexin-1 modulates T-cell activation and differentiation. Blood.

[25] Shin H.M., Kapoor V.N., Kim G., Li P., Kim H.R., Suresh M., Kaech S.M., Wherry E.J., Selin L.K., Leonard W.J. (2017). Transient expression of ZBTB32 in anti-viral CD8^+^ T cells limits the magnitude of the effector response and the generation of memory. PLoS Pathog..

[26] Takeuchi A., Itoh Y., Takumi A., Ishihara C., Arase N., Yokosuka T., Koseki H., Yamasaki S., Takai Y., Miyoshi J. (2009). CRTAM confers late-stage activation of CD8^+^ T cells to regulate retention within lymph node. J. Immunol..

[27] Lemieux A., Sannier G., Nicolas A., Nayrac M., Delgado G.G., Cloutier R., Brassard N., Laporte M., Duchesne M., Sreng Flores A.M. (2024). Enhanced detection of antigen-specific T cells by a multiplexed AIM assay. Cell Rep. Methods.

[28] Brunet-Ratnasingham E., Morou A., Dubé M., Niessl J., Baxter A.E., Tastet O., Brassard N., Ortega-Delgado G., Charlebois R., Freeman G.J. (2022). Immune checkpoint expression on HIV-specific CD4^+^ T cells and response to their blockade are dependent on lineage and function. EBioMedicine.

[29] Niessl J., Baxter A.E., Morou A., Brunet-Ratnasingham E., Sannier G., Gendron-Lepage G., Richard J., Delgado G.G., Brassard N., Turcotte I. (2020). Persistent expansion and Th1-like skewing of HIV-specific circulating T follicular helper cells during antiretroviral therapy. EBioMedicine.

[30] Morou A., Brunet-Ratnasingham E., Dube M., Charlebois R., Mercier E., Darko S., Brassard N., Nganou-Makamdop K., Arumugam S., Gendron-Lepage G. (2019). Altered differentiation is central to HIV-specific CD4^+^ T cell dysfunction in progressive disease. Nat. Immunol..

[31] Fu N., Xie F., Sun Z., Wang Q. (2021). The OX40/OX40L Axis Regulates T Follicular Helper Cell Differentiation: Implications for Autoimmune Diseases. Front. Immunol..

[32] Zaric M., Marini A., Nielsen C.M., Gupta G., Mekhaiel D., Pham T.P., Elias S.C., Taylor I.J., de Graaf H., Payne R.O. (2021). Poor CD4^+^ T Cell Immunogenicity Limits Humoral Immunity to P. falciparum Transmission-Blocking Candidate Pfs25 in Humans. Front. Immunol..

[33] Nielsen C.M., Ogbe A., Pedroza-Pacheco I., Doeleman S.E., Chen Y., Silk S.E., Barrett J.R., Elias S.C., Miura K., Diouf A. (2021). Protein/AS01(B) vaccination elicits stronger, more Th2-skewed antigen-specific human T follicular helper cell responses than heterologous viral vectors. Cell Rep. Med..

[34] Croft M., So T., Duan W., Soroosh P. (2009). The significance of OX40 and OX40L to T-cell biology and immune disease. Immunol. Rev..

[35] Sakkestad S.T., Steinsland H., Skrede S., Lillebø K., Skutlaberg D.H., Guttormsen A.B., Zavialov A., Paavilainen S., Søyland H., Sævik M. (2019). A new human challenge model for testing heat-stable toxin-based vaccine candidates for enterotoxigenic Escherichia coli diarrhea—Dose optimization, clinical outcomes, and CD4^+^ T cell responses. PLoS Negl. Trop. Dis..

[36] Brezar V., Hani L., Surenaud M., Hubert A., Lacabaratz C., Lelièvre J.D., Levy Y., Seddiki N. (2017). Negative modulation of suppressive HIV-specific regulatory T cells by IL-2 adjuvanted therapeutic vaccine. PLoS Pathog..

[37] Brezar V., Ruffin N., Richert L., Surenaud M., Lacabaratz C., Palucka K., Thiébaut R., Banchereau J., Levy Y., Seddiki N. (2015). Decreased HIV-specific T-regulatory responses are associated with effective DC-vaccine induced immunity. PLoS Pathog..

[38] Winkler F., Hipp A.V., Ramirez C., Martin B., Villa M., Neuwirt E., Gorka O., Aerssens J., Johansson S.E., Rana N. (2023). Enolase represents a metabolic checkpoint controlling the differential exhaustion programmes of hepatitis virus-specific CD8^+^ T cells. Gut.

[39] Wildner N.H., Walker A., Brauneck F., Ditt V., Peine S., Huber S., Haag F., Beisel C., Timm J., Schulze Zur Wiesch J. (2022). Transcriptional Pattern Analysis of Virus-Specific CD8^+^ T Cells in Hepatitis C Infection: Increased Expression of TOX and Eomesodermin During and After Persistent Antigen Recognition. Front. Immunol..

[40] Mazouz S., Salinas E., Bédard N., Filali A., Khedr O., Swadling L., Abdel-Hakeem M.S., Siddique A., Barnes E., Bruneau J. (2022). Differential immune transcriptomic profiles between vaccinated and resolved HCV reinfected subjects. PLoS Pathog..

[41] Mazouz S., Boisvert M., Abdel-Hakeem M.S., Khedr O., Bruneau J., Shoukry N.H. (2021). Expansion of Unique Hepatitis C Virus-Specific Public CD8^+^ T Cell Clonotypes during Acute Infection and Reinfection. J. Immunol..

[42] Hartnell F., Esposito I., Swadling L., Brown A., Phetsouphanh C., de Lara C., Gentile C., Turner B., Dorrell L., Capone S. (2020). Characterizing Hepatitis C Virus-Specific CD4^+^ T Cells Following Viral-Vectored Vaccination, Directly Acting Antivirals, and Spontaneous Viral Cure. Hepatology.

[43] Chen D.Y., Wolski D., Aneja J., Matsubara L., Robilotti B., Hauck G., de Sousa P.S.F., Subudhi S., Fernandes C.A., Hoogeveen R.C. (2020). Hepatitis C virus-specific CD4^+^ T cell phenotype and function in different infection outcomes. J. Clin. Investig..

[44] Schulze Zur Wiesch J., Ciuffreda D., Lewis-Ximenez L., Kasprowicz V., Nolan B.E., Streeck H., Aneja J., Reyor L.L., Allen T.M., Lohse A.W. (2012). Broadly directed virus-specific CD4^+^ T cell responses are primed during acute hepatitis C infection, but rapidly disappear from human blood with viral persistence. J. Exp. Med..

[45] Lucas M., Ulsenheimer A., Pfafferot K., Heeg M.H., Gaudieri S., Gruner N., Rauch A., Gerlach J.T., Jung M.C., Zachoval R. (2007). Tracking virus-specific CD4^+^ T cells during and after acute hepatitis C virus infection. PLoS ONE.

[46] Lokhande M.U., Thimme R., Klenerman P., Semmo N. (2015). Methodologies for the Analysis of HCV-Specific CD4^+^ T Cells. Front. Immunol..

